# A framework for developing and evaluating digital and public health tools

**DOI:** 10.1093/eurpub/ckac129.150

**Published:** 2022-10-25

**Authors:** B Schüz, S Müllmann, C-C Pan, T Jahnel, S Forberger, D Jürgens, N Pedros Barnils, A Gerhardus

**Affiliations:** 1 Institute for Public Health and Nursing Science, University of Bremen, Bremen, Germany; 2 Leibniz ScienceCampus Digital Public Health B, Bremen, Germany; 3 BIPS, Bremen, Germany

## Abstract

To paraphrase a classic, evaluating digital technologies in health is a bit like eating spinach - no one is against it in principle because it is good for you. However, no one would do it unless being asked to. In recent years, the sheer number of digital health technologies that potentially fulfil public health purposes has increased tremendously. The basis for evaluating such tools for public health purposes however has not met this pace, and in particular frameworks for the systematic development and evaluation of digital technologies in public health are rare. Existing frameworks for digital technologies focus on clinical aspects of digital health applications (e.g., NICE Evidence standards framework for digital health technologies), thus lacking both a population and prevention focus. Generic frameworks such as the Health Technology Assessment (HTA) methodology do not contain items specific to digital technologies and public health purposes. Here, we describe the process of developing a framework specific for the development and evaluation of digital public health technologies based on the core HTA model. We conduct a scoping review of frameworks for the development and the evaluation of technologies in public health and digital health, following PRISMA-SCR guidelines. The identified frameworks are then mapped onto the core HTA model to develop additional items specific for the development and the evaluation of digital technologies in public health. These additional items can be used to integrate the development and evaluation of digital technologies for public health purposes within the wider HTA context, making this process both transferable and scalable.

